# Temperature warming strengthens the mutualism between ghost ants and invasive mealybugs

**DOI:** 10.1038/s41598-017-01137-0

**Published:** 2017-04-19

**Authors:** Aiming Zhou, Xiaobin Qu, Lifan Shan, Xin Wang

**Affiliations:** Hubei Insect Resources Utilization and Sustainable Pest Management Key Laboratory, College of Plant Science and Technology, Huazhong Agricultural University, Wuhan, 430070 China

## Abstract

Although the exogenous forces that directly affect the mutualisms between ants and honeydew-producing hemipterans have been well documented, few studies have been focused on the impacts of environmental warming on ant-hemipteran interactions. Here, we investigated how temperature warming affects the mutualism between ghost ant *Tapinoma melanocephalum* and invasive mealybug *Phenacoccus solenopsis* by experimental manipulation of temperature. We found that higher temperatures have significant direct effects on the growth rate of mealybug colony, and the positive effect of ant tending on mealybug colony growth is temperature-dependent. Honeydew excretion by mealybugs was affected by ant tending and temperature warming, and was significantly increased under higher temperature. The effect of ant tending on percentage parasitism was also influenced by temperature warming. Ant performance including tending level, aggression, activity, and honeydew consumption was enhanced by temperature warming, which may provide superior protection to the mealybugs. Our results show that ghost ant-mealybug mutualism is strengthened in a warmer environment. These findings may facilitate the prediction of how each partner in the ant-hemipteran-enemy interactions responds to increasing temperature.

## Introduction

Global mean surface air temperature has been increased by approximately 0.5 °C in the 20^th^ century and will be further elevated by 1.7–4.9 °C during 1990 to 2100^1–3^. Temperature warming is beginning to have profound effects on the ecosystems^4^. Temperature changes not only directly affect the physiology and behavior of the focal species, but also indirectly influence other species with which the focal species interacts^5^. As poikilotherms, insects appear to quickly respond to the increase of ambient temperature^6^. Climate warming directly affects the development, survival, distribution and abundance of herbivores^7–9^. Usually, elevated temperature increases insect overwintering, damage magnitude and range expansion^10^. Besides, climate change may significantly modify the dynamic interactions between plants and insects^11^. Temperature increase may facilitate the growth of herbivores through restraining the concentrations of several phenolic compounds in plant leaves^12^.

Global climate changes are significantly altering multi-species interactions and the structure of ecological communities. Most of the studies have been focused on the effects of increase in CO_2_, which can directly affect plant physiology and indirectly alter the interactions among plants, herbivores and pathogens^13^. Currently, the effects of CO_2_ increase on insect feeding behaviors^14^, phytohormone-mediated defenses^14, 15^, and secondary metabolite-mediated resistance^13, 16, 17^ have been clearly elucidated. In contrast, the effect of temperature warming on multiple trophic levels has received relatively less attention. Environmental temperature can regulate a number of important biological processes and species interactions. Temperature changes can alter the consumption, growth and survival of insect herbivores, suggesting that temperature-driven impacts on herbivory could influence plant community composition or nutrient cycling^18^. However, there have been few studies addressing the effects of temperature rising on the interspecies interaction at the community level so far.

Reciprocally beneficial interaction between ants and hemipterans is a common phenomenon which plays key roles in the functioning of ecosystems^19–21^. This mutual interaction can be strongly influenced by the local neighborhood in which it occurs. The benefits provided by one mutualist species to another can vary along with the changes in biotic factors, such as the density of associated species, seasonality, spatial distribution, identity of species involved and quality of host plants^22–27^. In contrast, there have been very few studies about the direct effects of abiotic factors on ant-hemipteran-enemy-host plant interaction patterns. Multiple abiotic factors often influence the interspecies interactions and species coexistence patterns. For instance, temperature variation may modify both ant foraging behavior and aggression, thereby changing the competitive interactions and species co-occurrence patterns^28^. Increase of environmental temperature can promote the ingestion dynamics through directly affecting the sucking pump muscles in the nectarivorous ants *Camponotus mus* ^29^. Surface temperature is the main regulatory factor that determines the departure rate of harvester ants *Messor barbarus*, and the activity of worker ants is positively correlated with surface temperature^30^. Some ant species forage more frequently and recruit foragers faster under warmer conditions^31^. Moreover, ant foraging frequency mediates the tending level and protection ability for hemipterans^32, 33^. Benefits to hemipterans are positively and linearly related to ant tending level^26, 34, 35^. Aphids directly respond to elevated temperature with decreased developmental time and reduced adult and progeny weight^36, 37^. Pea Aphid, *Acyrthosiphon pisum*, ingested fluid from sieve element cells less frequently when exposed to extreme temperatures (such as 5 °C and 35 °C) than when exposed to other temperatures. Aphids at optimal temperatures (25–30 °C) ingested fluid from sieve elements for a longer period, probed for a longer time, and produced more offspring than those under other temperatures^38^, suggesting that probing behavior of aphids is enhanced under temperature warming. Besides, plant volatile emissions induced by herbivory are quantitatively related to the degree of herbivore ingestion and the extent of damage^39, 40^, and these varied volatiles play key roles in the host location of predators and parasitoids^41^. These previous studies suggest that temperature increase can considerably influence the concerned species and may bring profound chain effects to the interaction species. However, most of these studies only separately explored the effects on a single species in different research systems, and there have been very few studies dealing with the chain effects on related species and their interspecific interactions in the same research system. The integrated effects of temperature increase on the complex interactions among multiple trophic levels remain largely unknown.

The mutualisms between ants and hemipterans have been well documented. Ant tending improves the survival and colony growth of hemipterans, primarily by protecting the hemipterans from the attack by natural enemies. As a return, the ants receive large amounts of honeydew in a consumer-resource mutualism^19, 42–46^. This mutual relationship is stable when the trade-off between the cost and benefit is in balance^47^. Currently, many studies have documented that these steady interactions are mediated by numerous biotic factors^23, 34, 35, 48–53^. Both mealybug *Phenacoccus solenopsis* Tinsley (Hemiptera: Pseudococcidae) and ghost ant *Tapinoma melanocephalum* (Fabricius) (Hymenoptera: Formicidae) are important invasive species in China, which have established a close mutualistic relationship in the ecosystem^54^. Our previous study also showed that the mutualism between ghost ants and invasive mealybugs is significantly affected by the quality of host plant^26^. In contrast, few studies have reported the effects of temperature warming on ant-hemipteran-natural enemy interactions. Understanding how each partner in the ant-hemipteran-enemy interactions responds to changing temperature is critical to the clarification of the important ecological interactions mediated by climatic changes. We hypothesized that temperature warming would positively affect ghost ant-mealybug mutualism. In this study, we tested how temperature warming affects ant-hemipteran-parasitoid interactions, using the interactions among ghost ant *T*. *melanocephalum*-invasive mealybug *P*. *solenop*sis- aggressive parasitoid *Aenasius bambawalei* as a model system. As a result, the effects of temperature warming on the benefits to the mealybug, parasitism, ant performance, and integrated effects on ghost ant-mealybug mutualism were clarified.

## Materials and Methods

### Plants and Insects

Cotton plants were cultivated in plastic flowerpots (18 cm × 14 cm × 17 cm) in greenhouse. Each plant was approximately 35–40 cm in height and had 15 true leaves. Colonies of *P*. *solenopsis* were collected from the experimental field of Huazhong Agricultural University (HZAU, Wuhan, Hubei province, China) and reared on cotton plants in the laboratory. The 1^st^ instar mealybug nymphs were inoculated on the cotton and raised for 4–5 generations. The mealybug colonies were reared in greenhouse (temperature, 28 ± 1 °C; relative humidity, 60–70%; under an LD: 16:8 h photocycle). Colonies of ghost ants were collected from the suburb of Wuhan. The colonies were separated into a number of subcolonies. Each subcolony included one queen, adult workers (approximately 1500 individuals), pupae, larvae, and eggs. The ants were placed in plastic petri dish with plaster (6 cm in diameter), which served as an artificial nest. Ant subcolonies were maintained with distilled water plus a 10% honey solution, which was distributed through tubes. The parasitoid *A*. *bambawalei* was collected from the cotton field of HZAU. The parasitoid arrived as mummified mealybugs (parasitized by *A*. *bambawalei*) and separated into gel capsules (10 mm in length) until adult emergence. After emergence, the wasps were randomly paired and allowed to copulate. Copulation was observed in all pairings, and the fertilized female wasps were used in the experiments 24 h after the initial pairing. The larvae of the predator *Harmonia axyridis* were collected from cotton in the field. The ladybeetles were fed with mealybug nymphs in the laboratory at 28 ± 1 °C and maintained under an LD 16:8 h photocycle.

## Experimental Design

### Effects of ant tending, parasitoid and variation in temperature on the benefits to mealybugs

A full-factorial 3-way design was used in the experiment following the protocol of Barton and Ives^5^. The effects of ant tending, parasitoid and increasing temperature on mealybug colony growth were observed and measured. There were 16 combinations of ant tending (present/absent) × parasitoids (present/absent) × temperature (23 °C/26 °C/29 °C/32 °C). Each combination was repeated 12 times. We transferred 60 2^nd^ instar mealybugs to cotton plants and allowed them to acclimate and feed for 24 h. Each flowerpot was placed in a plastic container (50 × 40 × 16 cm) and covered by a cage with nylon netting (70 × 70 × 100 cm).

A subcolony of ghost ants was placed in each plastic flowerpot, and then the ants established new nests in the soil immediately. Worker ants could travel to forage for food through the plant trunk. The ants were given two mealworms and water (50 mL) every two days. After 24 h, two fertilized female parasitoids were placed on each selected plant. The experiments lasted for 8 weeks. The numbers of surviving and mummified mealybugs and the number of tending ants on the plant were counted every week. The effects of ant tending, parasitoid and increasing temperature on the colony growth rate of mealybugs were analyzed. The colony growth rate of mealybugs was defined as the final population density divided by the initial population density. The parasitism rate was defined as the number of mummified mealybugs divided by the total number of mealybugs (mummified and surviving mealybugs) on each plant. The mummified mealybugs were easily identified by the decreasing wax and the body color of the mealybugs. We defined the ant tending level as the mean number of tending ants per mealybug.

### Effects of temperature warming on ant aggression

Four temperature treatments (23 °C/26 °C/29 °C/32 °C) were also used in this experiment. Each combination was replicated 15 times. We transferred 60 2^nd^ instar mealybugs to each individual cotton plant. After 24 h, two ladybeetle larvae were placed on the plants as predators. As previously described, a subcolony of ghost ants was placed in each plastic flowerpot after another 24 h. We recorded the time it took for an ant to make physical contact with the ladybeetle. We also observed the ant activities under different temperature treatments. Ant foraging activity on the plants was determined by counting the number of foraging ants moving up and down the plant trunk for 3 min.

### Effects of ant tending and temperature warming on honeydew excretion by mealybugs

In this experiment, we tested the effects of temperature warming on the weight of honeydew drops excreted by mealybugs. This experiment also crossed two ant treatments (ant tending and no ant) with four temperature treatments (23 °C/26 °C/29 °C/32 °C). There were 8 combinations and each combination was repeated 6 times. Sixty 3^rd^ instar mealybugs were transferred to cotton plant. Ant colonies were placed in plastic flowerpots after 24 h. We sampled the honeydew present on the anus of the mealybugs and cotton plant using filter papers, which had been pre-weighed and dried off at 40 °C for 24 h. Honeydew was sampled at one hour intervals for 6 h (from 9 a.m. to 3 p.m.) for one week. All honeydew-adsorbed filter papers were re-weighed using microbalance (Sartorius, BS, 224 S). The honeydew available per mealybug was determined as the average difference in the mass of the filter papers before and after the adsorption of honeydew. The total mass of honeydew was calculated when the collections were finished.

### Effects of temperature warming on honeydew consumption by ghost ants

We also determined the consumption of honeydew by ghost ants under the temperature gradient (23 °C/26 °C/29 °C/32 °C). Sixty 3^rd^ instar mealybugs were transferred to cotton plant. Ant colony was placed in each plastic flowerpot after 24 h. We weighed 30 ants exiting the ant nest and moving towards the mealybugs (traveling up) and another 30 ants that tended the mealybugs or fed on honeydew and carried honeydew away towards the ant nest (traveling down) by using a microbalance (Sartorius, BS, 224 S). The honeydew consumption by ghost ants was calculated as the difference in mean weights between the two ant groups. Each treatment was repeated 12 times.

### Statistical Analyses

To satisfy the preconditions of variance analysis, the data were transformed. The growth rate of the mealybug colony, ant attack time and ant activity were log-transformed; the percentage of parasitism on the mealybugs was treated by the arcsine square root-transformation; and the ant tending level was treated by the square root-transformation. When the data were normally distributed and had similar variances, an analysis of variance (ANOVA) using the Type III sum of squares was performed to compare the means among all measured variables. When the ANOVA results were significant, multiple comparisons of means were performed with Tukey HSD post-hoc analysis. Paired sample t-test was performed to analyze the difference in weights between traveling-down and traveling-up ants. We used the non-parametric Kruskal-Wallis test to compare the median of honeydew consumption by ants under different temperature treatments, and also the Mann-Whitney test (or the two-sample Kolmogorov-Smirnov test) was performed for multiple comparisons among different groups if the results of the Kruskal-Wallis test showed significant differences at 0.05 significance level. All statistical analyses were conducted using SPSS version 14.0 (SPSS Inc., Chicago, IL).

## Results

### Colony growth of mealybugs

**Figure 1:**
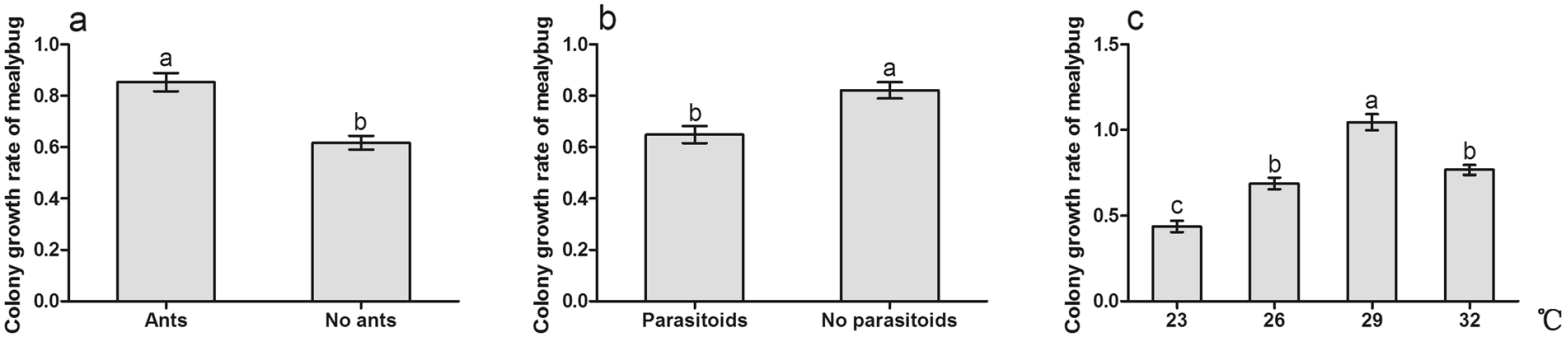
Effect of ant tending, the presence of parasitoids, temperature warming on mealybug colony growth. (**a**) Ant tending; (**b**) Parasitoid; (**c**) Temperature. Results are expressed as the means ± SE. Bars sharing the same letters indicate no significant differences between the treatments (*P* > 0.05).

Ant tending, parasitoid, temperature and the interactions between ant tending and temperature showed significant effects on mealybug colony growth (Table 1: Ants, Parasitoids, Temperature, Ants × Temperature). The growth rate was obviously improved by ant tending (Table 1: Ants, Fig. 1a); in contrast, it was notably decreased by the presence of parasitoids (Table 1: Parasitoids, Fig. 1b). The growth rate of mealybug colony at 29 °C was higher than that under other temperature treatments (Fig. 1c). No significant interactions were found for ant tending and parasitoids, parasitoids and temperature, nor for all three factors together (Table 1).

**Table 1:** Analysis of variance of the mealybug colony growth rate with ant tending, parasitoid, and temperature.

Source of variation	Sum of Squares	df	Mean Square	F	Sig.
Ants	2.685	1	2.685	71.777	0.000
Parasitoids	1.425	1	1.425	38.096	0.000
Temperature	8.980	3	2.993	80.025	0.000
Ants × Parasitoids	0.000	1	0.000	0.010	0.922
Ants × Temperature	1.040	3	0.347	9.264	0.000
Parasitoids × Temperature	0.074	3	0.025	0.657	0.580
Ants × Parasitoids × Temperature	0.029	3	0.010	0.257	0.856

### Parasitism of mealybugs

**Figure 2:**
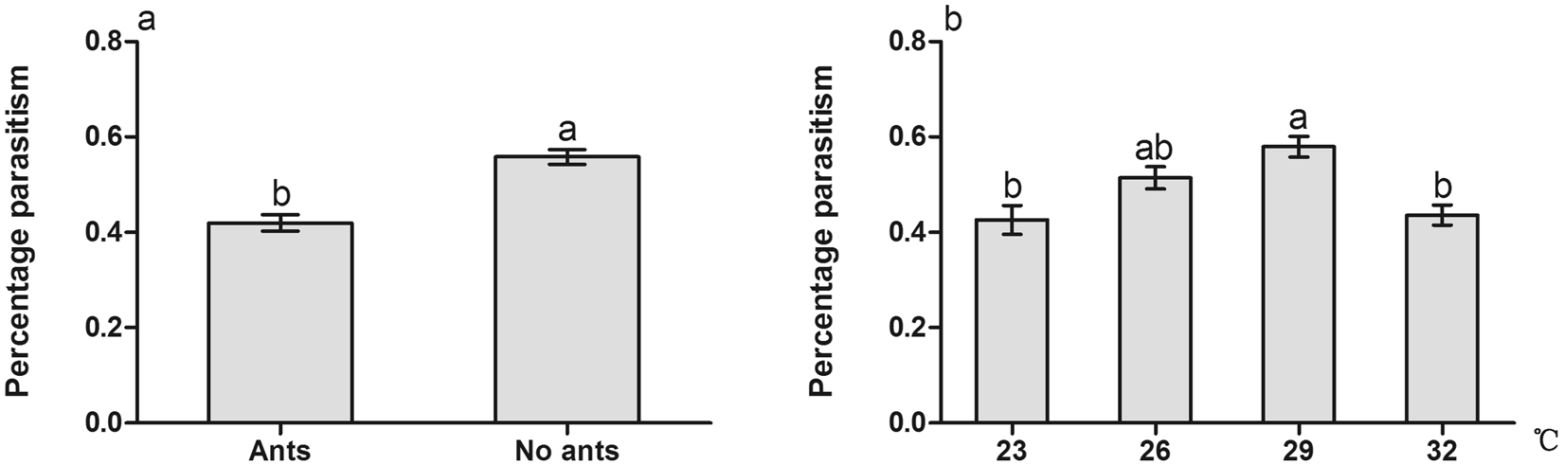
Effect of ant tending, the presence of parasitoids, and temperature warming on the percentage of parasitism. (**a**) Ant tending; (**b**) Temperature. Results are expressed as the means ± SE. Bars sharing the same letters indicate no significant differences between the treatments (*P* > 0.05).

Ant tending, temperature and the interactions between ant tending and temperature also had significant effects on the percentage parasitism of mealybugs (Table 2: Ants, Temperature, Ants × Temperature). Parasitism significantly decreased with ant tending (Table 2: Ants, Fig. 2a). Percentage parasitism at 29 °C was significantly higher than that under 23 °C and 32 °C (Table 2: Temperature, Fig. 2b).

**Table 2:** Analysis of variance of the percentage parasitism with ant tending and temperature.

Source of variation	Sum of Squares	df	Mean Square	F	Sig.
Ants	0.458	1	0.458	54.889	0.000
Temperature	0.373	3	0.124	14.891	0.000
Ants × Temperature	0.128	3	0.043	5.103	0.003

### Ant tending level

**Figure 3:**
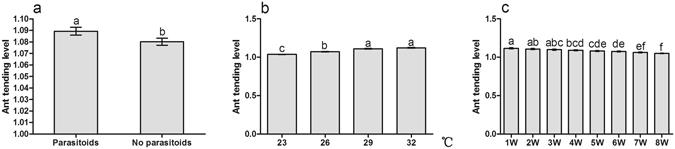
Effect of the presence of parasitoids, temperature warming, and investigation date on ant tending level. (**a**) Parasitoid; (**b**) Temperature; (**c**) Investigation date. Results are expressed as the means ± SE. Bars sharing the same letters indicate no significant differences between the treatments (*P* > 0.05).

The results showed that parasitoids, temperature and investigation date had significant effects on ant tending level (Table 3: Parasitoids, Temperature, Time). Ant tending level was higher under parasitic pressure than under no parasitoids (Fig. 3a), and was increased with temperature rising. Tending level at 29 °C and 32 °C was significantly higher than that at 26 °C and 23 °C (Fig. 3b). In contrast, it showed a notable decrease along with the increase in treatment time (Fig. 3c). Interactions between temperature and investigation date on ant tending level were significant (Table 3 Temperature × Time). No significant interactions were found for parasitoids and temperature, parasitoids and investigation date, nor for all three factors together (Table 3).

**Table 3:** Analysis of variance of the ant tending level with parasitoid, temperature, and investigation date.

Source of variation	Sum of Squares	df	Mean Square	F	Sig.
Parasitoids	0.016	1	0.016	6.341	0.012
Temperature	0.879	3	0.293	116.040	0.000
Time	0.334	7	0.048	18.903	0.000
Parasitoids × Temperature	0.002	3	0.001	0.234	0.873
Parasitoids × Time	0.002	7	0.000	0.099	0.998
Temperature × Time	0.094	21	0.004	1.772	0.018
Parasitoids × Temperature × Time	0.006	21	0.000	0.106	1.000

### Ant aggression

**Figure 4:**
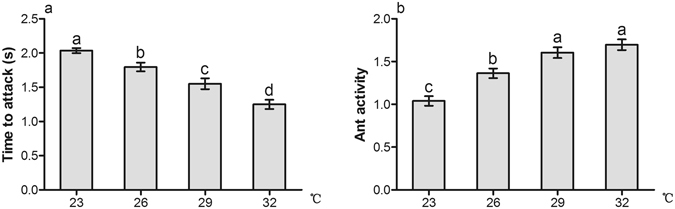
Effect of temperature warming on ants attacking ladybeetles. Results are expressed as the means ± SE. Bars sharing the same letters indicate no significant differences between the treatments (*P* > 0.05).

Temperature warming improved the aggression and activity of ghost ants. There were significant differences in ant aggression among different temperature treatments (F = 27.709, *df* = 3, *P* < 0.001). Ants could make physical contact with the ladybeetle more quickly with temperature warming, and were more aggressive towards the intruding predators at 32 °C compared with at other temperatures (Fig. 4a). There were also significant differences in ant activity at different temperatures (F = 24.617, *df* = 3, *P* < 0.001). Ant foraging frequency at 29 °C and 32 °C was higher than that at 26 °C and 23 °C, while was not significantly different between 29 °C and 32 °C (Fig. 4b).

### Honeydew production by mealybugs

**Figure 5:**
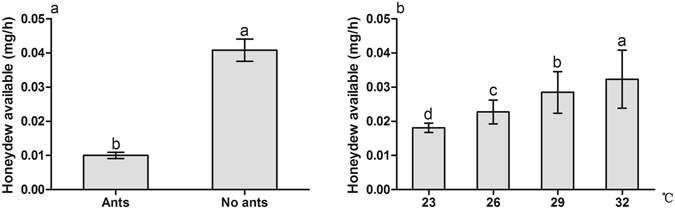
Effect of ant tending and temperature warming on the available honeydew mass excreted by mealybug. (**a**) Ant tending; (**b**) Temperature. Results are expressed as the means ± SE. Bars sharing the same letters indicate no significant differences between the treatments (*P* > 0.05).

Honeydew excretion per mealybug was significantly affected by ant tending, temperature warming and their interactions (Table 4). Ants removed most of the honeydew available per mealybug in comparison with no-ant treatment (Fig. 5a). Along with the temperature gradients, honeydew excretion per mealybug followed the order of 32 °C > 29 °C > 26 °C > 23 °C, and all the differences reached significant level (Fig. 5b).

**Table 4:** Analysis of variance of the available honeydew mass excreted by mealybug with ant tending and temperature.

Source of variation	Sum of Squares	df	Mean Square	F	Sig.
Ants	0.011	1	0.011	665.291	0.000
Temperature	0.001	3	0.000	28.754	0.000
Ants × Temperature	0.004	3	0.001	79.736	0.000

### Honeydew consumption by ghost ants

**Figure 6:**
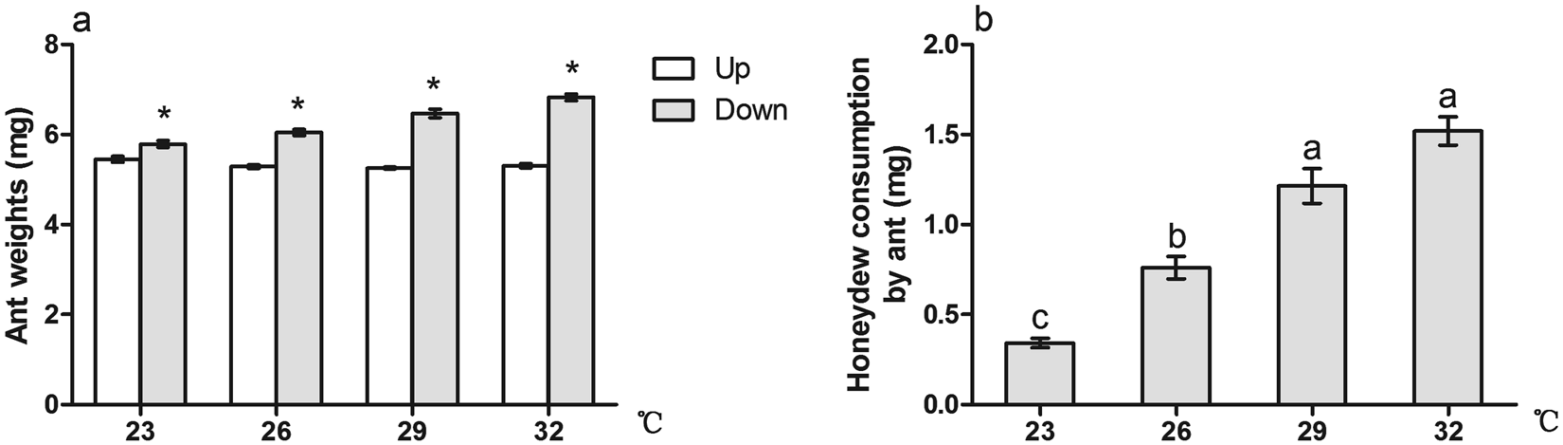
Effect of temperature warming on the honeydew consumption by ant. (**a**) Difference of ant weights between traveling down and traveling up; (**b**) Temperature. Results are expressed as the means ± SE. Asterisk (*) on bars indicate significant differences in the ant weights between travel up and travel down (*P* < 0.05). Bars sharing the same letters indicate no significant differences between the treatments (*P* > 0.05).

Ants traveling down weighed more than ants traveling up at each temperature (Fig. 6a, t = −13.124, *P* < 0.001; t = −12.452, *P* < 0.001; t = −12.569, *P* < 0.001; t = −19.380, *P* < 0.001). Honeydew consumption by ghost ants increased with increasing temperature. Ants harvested more honeydew at 32 °C and 29 °C than at 26 °C and 23 °C (Fig. 6b, *χ* ^2^ = 36.332, *df* = 3, *P* < 0.001).

## Discussion

The net effect of environmental perturbation on species in interconnected ecological communities directly or indirectly influences the interactions among species^5^. The interspecies interaction patterns in ecosystems are often mediated by multiple abiotic factors. Here, we observed the responses of each partner in ant-mealybug-parasitoid interactions under manipulated temperatures. The results show that temperature warming has positive effects on mealybug colony growth, honeydew excretion and ant performance, including tending level, aggression and activity. Although both the colony growth and parasitism of mealybugs decreased under extreme temperature (32 °C), ant tending level and aggression were greatly enhanced. It has been demonstrated that there is a positive linear relationship between ant tending level and mealybug colony growth rate^35^, which suggests that temperature warming could promote mealybug colony growth directly or indirectly. Thus, the net effect of temperature warming is to increase mealybug colony by benefiting ghost ant-mealybug mutualism.

Recent research has found that there is no effect of ant tending or combined effect of ant tending and temperature on the growth rate and instar production of scale insect *Eriococcus coriaceus*. Per capita growth rate of *E*. *coriaceus* was reduced with increasing temperature^46^. However, our results indicate that the colony growth of mealybugs is positively influenced by ghost ant tending, while is negatively affected by parasitoids, which is consistent with the findings in other studies^26, 35, 54^. Mealybugs showed a higher growth rate at higher temperature, but displayed an inhibited growth rate at extreme temperature (Fig. 1c). Prasad also reported that mealybugs showed higher fecundity at 25 °C–30 °C^55^. The high growth rate of *P*. *solenopsis* at higher temperature is not surprising because it exhibits strong adaptability in many habitats as an invasive species^56^. We also found that there are significant interactions between ant tending and temperature warming, indicating that the benefits from ghost ant tending are temperature-dependent. It has been suggested that benefits to hemipteran from ant tending are strongly related to the ant tending level^34, 35^. In this study, ant tending level was observably increased with temperature warming (Fig. 3b). Thus, mealybug colony growth may be suppressed directly by temperature warming, especially by extreme temperature, but improved indirectly by increased ant tending level. Protection level of ant tending on treehopper *Guayaquila xiphias* varies not only with time, but also with changes in the abundance of natural enemies^57^. We also found that ant tending level was higher with parasitoids than without parasitoids (Fig. 2a), indicating that the degree of the protection by ants may be stimulated by the harassment of parasitoids. In addition, ant tending level was decreased gradually with the extension of treatment time (Fig. 3c). We infer that increasing mealybug abundance is responsible for the decrease of tending level. We observed a positive correlation between ant foraging activity and the density of mealybugs on the plants, but the ant recruitment did not proportionally increase as mealybug abundance, suggesting that the ghost ant tending level is not improved as the mealybug density increases, which has also been concluded in previous studies^33–35^. It is clear that hemipterans benefit from ant tending through decreasing the performance of predators and parasitoids^53, 58, 59^. We also found that the percentage parasitism of mealybugs with ant tending was considerably lower than that of mealybugs without ant tending. In addition, the percentage parasitism varied with temperature warming: it exhibited a high level at 29 °C but declined at 32 °C (Fig. 2b). Previous studies also demonstrated the temperature-dependent parasitizing efficiency of parasitoids^60–63^. Although the optimal temperature for successful parasitism is various for different species, the proportion of successful parasitism increased at temperatures >15 °C but sharply declined at temperatures >30 °C^62, 63^.

Various factors can evoke or influence ant aggression, such as temperature and hunger^64, 65^. Foraging and activity of some subordinate ant species are considerably influenced by temperature, and the food resource possession and aggression of these ant species exhibit a positive relationship with increasing temperature^66^. We found that ghost ants were more aggressive, and moved more frequently at higher temperatures (Fig. 4a,b). By contrast, winter ants *Prenolepis imparis* are less aggressive towards predators and less abundant at higher temperatures^5^. We infer that the behavioral response patterns vary among different ant species. Increased ant aggression towards enemies at higher temperatures suppresses enemy performance, which facilitates the growth of mealybugs despite the lower potential colony growth rate at extreme temperatures. We also found significant effects of the interactions between ant tending and temperature on percentage parasitism, suggesting that inhibition of percentage parasitism by ant tending varies with temperature warming. Thus, ghost ant aggression and protection level are improved and mealybugs would be better protected with temperature warming, suggesting that ghost ant-mealybug mutualism will be strengthened in a warmer environment.

Several studies have indicated that the quality and quantity of the honeydew produced by hemipterans play an important role in mediating the stability and outcomes of hemipteran-ant interactions^19, 32, 67^. The most heavily attended aphid species produces the greatest volume of honeydew and the largest amount of total sugars and trisaccharides per unit time^67^. Our results show that honeydew production by mealybugs is largely influenced by ant tending, temperature warming and their interactions. Less honeydew was present in the treatment with ant tending than in the no-ant treatment (Fig. 5a), indicating that ants remove or consume some part of the honeydew. This is in accordance with the significant difference between ants in traveling-down and traveling-up groups (Fig. 6a). Both honeydew excretion by mealybugs and honeydew consumption by ants were increased with temperature warming (Figs 5b and 6b). Sagata and Gibb reported that honeydew production by scale insect *E*. *coriaceus* was significantly affected by ants, but not by temperature, and the weight of honeydew harvested by individual ants even decreased with increasing temperature^46^. They also suggested that *E*. *coriaceus* is likely to have the maximum ingestion at 23 °C; thus, increasing temperature does not result in greater ingestion of sap by *E*. *coriaceus* ^46^. Responses to increasing temperature of sap-sucking insects may vary among different species^36–38^. Proper temperatures may improve the flow of phloem sap by reducing sap viscosity or by increasing the turgor pressure of the plants^68^. Therefore, whether temperature warming has a positive or negative impact on ant-hemipteran mutualisms may be related to the variation characteristics of honeydew produced by hemipterans. Our findings demonstrate that temperature warming stimulates honeydew excretion by mealybugs and honeydew consumption by ants, suggesting that ghost ant-mealybug association is enhanced under temperature warming. In addition, honeydew quality may also mediate the ant-hemipteran mutualism^26, 67, 69^. Nutritional requirements of the ant colony and ant preference for sap-sucking insects may be influenced by the changes in quantity and quality of the honeydew. The nutritional contents of honeydew excreted by hemipteran can be directly affected by high temperature^70^. On the other hand, plant quality such as nutrients in the phloem may be changed by temperature warming. Although we did not test this possibility here, a previous study has suggested that sugar accumulation in the phloem elements may also be influenced by temperature warming^71^. Therefore, temperature warming may have indirect effects on ant-hemipteran mutualism by influencing the plant quality.

The negative impacts of temperature warming on ecosystems are receiving more and more attention in science and society. Our results suggest that temperature warming improves mealybug colony growth and ghost ant performance. Ghost ant-mealybug mutualism may be enhanced under temperature warming. Abundant populations of these two invasive species are present in various ecosystems in China, and they have been observed to cause serious damage to agriculture and forestry production. Previous studies have shown that the mutualism between the two species promotes the invasion by each species^26, 35, 54, 72^. Our findings indicate that the invasion of these two species may be further exacerbated due to the enhanced mutual association by temperature warming at least in this system. These findings also allow us to predict how each partner in the ant-hemipteran-enemy interactions may respond to increasing temperature.
